# Improving the efficiency of the regulatory approvals of HIV *in vitro* diagnostics in low- and middle-income countries: Perspective of the IAS Industry Liaison Forum diagnostic partners

**DOI:** 10.1371/journal.pgph.0004169

**Published:** 2025-04-10

**Authors:** Tammy Steuerwald, Duncan Blair, Elliot Cowan, Michael Isbell, Samuel Boova S., Julieta Firmat, Nelli Barriere, Roger Tatoud

**Affiliations:** 1 Regulatory Policy, Global Quality and Regulatory Division, Roche Diagnostics, Indianapolis, Indiana, United States of America; 2 Abbott Laboratories, Abbott Park, Illinois, United States of America; 3 Partners in Diagnostics, LLC, Rockville, Maryland, United States of America; 4 Independent Consultant, New York, New York, United States of America; 5 Medical and Scientific Affairs, Beckman Coulter Life Sciences, Miami, Florida, United States of America; 6 HIV Programmes and Advocacy, International AIDS Society, Geneva, Geneva, Switzerland; 7 Development and Partnerships, International AIDS Society, Geneva, Geneva, Switzerland; 8 Origena Consulting, Ferney Voltaire, France; McGill University, CANADA

## Abstract

*In vitro* diagnostics (IVDs) play a pivotal role in healthcare for the prevention, diagnosis, and treatment of infectious diseases, especially during epidemic and pandemic outbreaks. Improving access to IVDs holds promise for better healthcare outcomes, particularly in resource-limited settings. However, challenges persist, such as regulatory disparities and financial constraints, hindering broad access to IVDs. To address these challenges, the Industry Liaison Forum of the International AIDS Society convened regulatory agencies, industry stakeholders, and conducted research on IVD regulatory systems and practices in low- and middle-incomes countries. This paper synthesizes findings and makes recommendations from the Industry Liaison Forum diagnostic partners targeting key stakeholders to enhance regulatory capacity by embracing best practices and supporting initiatives improving diagnostic accessibility in resource-constrained settings. Converging regulations and reliance on internationally recognized approvals and standards can expedite access to diagnostics, including HIV diagnostics and monitoring tests without sacrificing quality or safety. This ongoing effort demands sustained engagement and collaboration. While challenging, improved regulatory frameworks promise significant benefits for global public health and end users. By fostering collaboration between industry, regulatory bodies, and other stakeholders, the Industry Liaison Forum aims to support a global effort to streamline regulatory processes, ultimately reducing barriers to accessing IVDs worldwide.

## Introduction

*In vitro* diagnostics (IVDs), including those for self-testing and point-of-care (PoC), play a crucial role in numerous aspects of healthcare, including prediction, prevention, diagnosis, case management, monitoring, treatment and containment, especially during an outbreak or pandemic [1]. As noted by the World Health Organization (WHO), conditions cannot be diagnosed and treated without medical devices (MDs), including IVDs [2]. Increasing access to IVDs has the potential to significantly improve healthcare outcomes, particularly for people living in countries with limited healthcare resources [3]. Unfortunately, many low- and middle-income countries face challenges in accessing diagnostics due to such factors as their healthcare settings, vulnerability of populations, and inadequate financing to meet costs [4] but also human resourcing, domestic policy development and implementation challenges. The challenges, including regulatory divergence, have long been recognized [3,5] and were formally acknowledged in 2023 when the 76th World Health Assembly approved a resolution on strengthening diagnostic capacity [6].

Considering these challenges and the need for solutions, the Industry Liaison Forum (ILF) of the IAS - International AIDS Society decided to leverage its membership to better understand the regulatory challenges of bringing quality IVDs to low- and middle-income countries and explore potential actions for improving access to diagnostic tools. The ILF was established in 2001 to promote and facilitate the full contribution of the biomedical industry to the global HIV response. It does this by catalysing multi-stakeholder dialogue, engagement and action to address barriers along the HIV prevention, diagnosis and care continuum. Its members include representatives of industry (originator and generic biopharmaceutical, diagnostic and other health-technology companies) and non-industry organizations (see Table 1). This paper summarizes insights and perspectives intended to advance the efficiency of regulatory approvals for IVDs worldwide, including IVD PoC diagnostics, and to provide support for the global efforts aimed at enhancing the development and accessibility of diagnostics in low- and middle-income countries. While many hurdles exist when it comes to accessing diagnostics, regulatory convergence stood out as one area that could significantly improve timely and equitable access across different jurisdictions.

**Table 1:** Industry Liaison Forum Roundtable - 24 February 2022 - End-to-end diagnostics implementation: Advocating for innovative solutions.

Topics	Contributors
Challenges and opportunities for IVD manufacturers – COVID-19 lessons learned Challenges and opportunities for IVD manufacturers in Southeast Asia (SEA) – COVID-19 lessons learned Challenges and opportunities for IVD manufacturers in South Africa - COVID-19 lessons learned Generalizable lessons from the UK response to COVID-19 Addressing the data gap to facilitate the regulation and uptake of IVD Overview of the WHO PQ for in vitro diagnostics	Tammy Steuerwald, Roche Diagnostics, USA. Kate Qi, SG Diagnostics, Singapore. Averouz Maritz, Medical Diagnostech, South Africa. Mike Messenger, Medicines and Healthcare products Regulatory Agency, UK. Anafi Mataka, African Society for Laboratory Medicine, Lesotho. Susie Braniff, In vitro Diagnostics Assessment, Regulation and Prequalification Unit, World Health Organization, Switzerland.
**Key Informant Interviews 2023**	
**Industry**	**Non-Industry**
Omega Diagnostics Group Partners in Diagnostics Beckman Coulter Life Sciences Roche Diagnostics	World Health Organization Elizabeth Glaser Pediatric AIDS Foundation Unitaid Food and Drugs Authority Ghana Pharmacy and Medicines Regulatory Authority (PMRA) - Malawi
**About the Industry Liaison Forum at the IAS**	
The Industry Liaison Forum, established in 2001, aims to enhance the biomedical industry’s role in the global HIV response. It fosters multi-stakeholder dialogue, engagement, and action to address challenges across the HIV prevention, diagnosis, and care continuum. Members include industry representatives, such as originator and generic biopharmaceutical companies, diagnostic firms, and other health technology providers, alongside non-industry organizations. Website: https://www.iasociety.org/industry-liaison-forum	
**Industry members**	**Non industry members**
Helen McDowell (Co-chair until July 2023) – ViiV Healthcare, UK Fernando Bognar (Co-chair since July 2023) – Gilead Sciences, USA Beverley Goede – Roche Molecular Systems, USA Elliot Cowan – Partners in Diagnostics, USA Ingrid Eshun-Wilsonova - Johnson & Johnson, South Africa James Rooney – Gilead Sciences, USA John Bannister – Accubio, UK Samuel (Tony) Boova – Beckman Coulter, USA Silas Holand – Merck, USA Tisha Wheeler – Roche Diagnostics, USA	Nittaya Phanuphak Pungpapong (Co-Chair) – Institute of HIV Research and Innovation in Bangkok, Thailand Boniface Dongmo Nguimfack – WHO HIV/AIDS Department, Switzerland Brent Allan – Australasian Society for HIV, Viral Hepatitis and Sexual Health Medicine (ASHM), IAS consultant, International Council of AIDS Service Organizations (ICASO), Australia Catherine Hankins – Amsterdam Institute for Global Health and Development (AIGHD) and McGill University, The Netherlands Carmen Perez Casas – Unitaid, Switzerland Colleen Daniels – Harm Reduction International, UK Anne Hoppe – Elizabeth Glaser Pediatric AIDS Foundation, USA Rahab Mwaniki – National Empowerment Network of People Living with HIV and AIDS in Kenya (NEPHAK), Kenya Ray Corrin – WHO PQ, Switzerland Sandra Nobre – Medicines Patent Pool, Switzerland Sandeep Juneja – TB Alliance, USA Wim Vandevelde – GNP+, South Africa Yodit Belew (since June 2021) – FDA, USA

## Methodology

The development of this paper followed a multi-phase approach combining stakeholder engagement, desk research, and expert consultations (Table 1). It builds on the methodology used for the 2018 publication by the ILF at the IAS, *The Manufacturers’ Perspective on World Health Organization Prequalification of In Vitro Diagnostics*, based on interviews with key industry stakeholders [7].

In response to the COVID-19 pandemic, the February 2022 roundtable *End-to-End Diagnostics Implementation: Advocating for Innovative Solutions* gathered experts to discuss region-specific challenges, opportunities, and regulatory implications for IVDs manufacturers. Insights from this event guided a desk review of regulatory systems and barriers in key countries. This review was complemented by interviews with representatives from regulatory and normative agencies, diagnostics manufacturers, and pharmaceutical industry stakeholders (see Table 1).

Rather than a survey of regulatory agencies, this study synthesizes insights from events, research, and consultations. It aims to amplify industry stakeholder perspectives and provide actionable recommendations to advance global regulatory harmonization.

### Regulatory challenges faced by manufacturers and distributors of IVDs in low- and middle-income countries

Regulatory approval is the gateway to accessing safe and effective diagnostics. However, at times, complexity and divergent regulatory frameworks can cause hurdles that manufacturers may choose to avoid. WHO has acknowledged that “costs of doing business, both direct (e.g., through paying user fees) and indirect (e.g., the regulatory burden of compliance with local requirements), may have an influence on whether medical devices are introduced to a particular market. If the costs of compliance appear disproportionately high compared with the potential of a market, or if regulatory requirements are not harmonized with those of other countries, manufacturers and importers may be discouraged from offering their products and that may impede achievement of national public health goals” [2].

Regulatory approval hurdles are especially acute in low- and middle-income countries, where there can be a lack of regulatory convergence to internationally recognized best practices and standards, as well as a significant lack of resources [5,8,9]. As a result, to ensure a feasible market access path, market hurdles in the absence of a publicly shared scientific rationale should be avoided.

Regulatory hurdles for IVDs exist in many forms:

- **Definition of an IVD.** Definitions are the backbone of regulation. Therefore, it is imperative to implement internationally consistent definitions of an medical devices (MD) and IVD. If a product is not regulated as an IVD in mature markets, an IVD designation in a low- or middle-income country may be seen as a hurdle that manufacturers choose to avoid. In this case, it is unlikely that the manufacturer followed quality management system (QMS) requirements specifically applicable to IVDs to develop and maintain the product. As a result, it would be going back to the drawing board to reverse engineer a product to fit the IVD requirements – likely seen as too costly an effort for market entry.
- **Risk classification**. Risk classification drives product requirements, including evidence and post-market surveillance. Therefore, divergence from the internationally recognized risk-classification scheme [10] can result in regulatory barriers that the manufacturer chooses to avoid. Regulatory controls should be proportionate to the level of risk associated with a device. For example, for lower-risk products, regulator review prior to marketing is not recommended [2]. However, some countries require all MD/IVDs, no matter their risk classification, to be reviewed in a way that is not proportional to risk (United Arab Emirates, Saudi Food and Drug Authority, Kuwait, Tanzania, Kenya, Egypt, Nigeria, Jordan, and Serbia). In contrast, regulators with a “mature” regulatory framework (for example, U.S. Food and Drug Administration, European Union, Singapore and Brazil) follow the risk-based approach and concentrate their reviews on higher-risk products while leveraging other methods, such as post-market controls, to monitor safety of lower-risk products. Such an approach is preferred as it expedites healthcare client access and conserves limited resources while protecting client safety. To assess the “maturity” level, WHO is using Global Benchmarking Tool (GBT) to evaluate regulatory authorities on a “scale of 1 (existence of some elements of regulatory system) to 4 (operating at advanced level of performance and continuous improvement)” [11].
- **Standards and Good Regulatory Practices (GRP)**. Local standards misaligned with internationally recognized standards (for example those of the International Organization for Standardization- ISO, and the International Electrotechnical Commission - IEC) may force manufacturers to redesign a product or perform time-consuming and expensive country-specific testing or conformity assessments. Not only is this a hurdle that manufacturers may choose to avoid, but it also runs counter to World Trade Organization obligations that require each member to use internationally recognized standards as the basis of their technical regulations to avoid barriers to trade. Implementation of GRP, as recommended by “mature regulators” and WHO, would prevent unnecessary misalignments that could block access. GRP is the quality control mechanism for the development of regulations, ensuring on a continuous and systematic basis that government rules are relevant, of the highest quality, cost effective, internationally aligned and least economically restrictive among alternatives with the same purpose [12].
- **Evidence requirements**. WHO does not recommend in-country clinical trials for every product unless there is a sound scientific reason. WHO recognizes that requiring in-country clinical trials can unnecessarily delay access. In-country clinical investigations (that is, systematic clinical investigation in the country in which market authorization is being sought) should not generally be a requirement. When adequate clinical evidence from another country, along with a clinical evaluation, has been provided to the national regulatory authority (NRA), as part of a market authorization application, a new in-country clinical investigation should not generally be required unless there is a compelling and sound scientific reason [2]. In all cases, informants acknowledged that where scientifically valid differences in requirements exist, time-limited testing or abridged testing may be needed and appropriate.
- **Dossier preparation**. Substantial effort and expense are required to assemble dossiers. The need to reformulate the same data repeatedly in different formats to meet the unique requirements of each individual NRA significantly increases the burden on diagnostic companies. The International Medical Device Regulators Forum (IMDRF) has a common dossier format for IVDs (and medical devices). Brazil, one of the IMDRF countries, established a reliance pathway and has aligned its dossier requirements to the IMDRF table of content [13]. In addition, the US and Canada launched the e-Star pilot [14] to identify differences in dossier requirements. Implementing the IMDRF dossier format not only reduces complexity for the manufacturer, but also makes it easier for one country to rely on another’s decisions because there is predictability in the review process and evidence requirements. One informant reported that due to the multiple steps required in one country, a new product might take as long as 36 months to get approved. Therefore, to ease unnecessary burdens and facilitate reliance, countries should strive to align with the IMDRF Table of Contents (TOC) and allow electronic submissions. As per WHO GMRF, “Regulatory authorities are encouraged to adopt such harmonized (e.g., IMDRF TOC) and electronic formats if they require submission of technical documentation. E-submission will enhance the exchange of documentation for regulatory reliance purposes.” [15].
- **Lot testing**. Blanket Lot testing is a long-standing practice (in both pre- and post-market testing) that is unique to low- and middle-income countries. While “mature regulators” reserve the right to test MD or IVDs (for example, in the event of evidence that suggests a safety concern), they do not perform testing on every, or even the majority of, MD or IVD lots – instead they take a risk-based approach. In addition, WHO does not recommend blanket lot testing for MD or IVDs: “In general, routine testing of medical devices by the NRA, either imported or locally produced, is not a cost-effective use of limited resources and is not recommended. The manufacturer has the primary responsibility for demonstrating that a device conforms to the essential principles of safety and performance, quality requirements, and all applicable national laws and regulations. Under the manufacturer’s quality management system, this includes any testing and documentation, all of which is subject to auditing and review by the NRA or CAB, either before market introduction or upon demand. As with all other evidence of conformity held or submitted by the manufacturer, that testing evidence is subject to review or audit/inspection by the regulatory authority” [15]. Further the WHO recommends that “some countries that have yet to implement effective regulation for medical devices but have a national industry or need to import high-risk (Class D) IVDs, may implement a system of risk based lot verification of such IVDs, pre-distribution to users or post distribution before they are put into service” [15]. The WHO also points out that, “the need for lot verification testing depends upon the other controls in place in the importing country and the extent of premarket evaluation conducted. Where there are stringent controls on transport and storage, and the receiving laboratory has in place an effective quality control programme that will detect problems in the performance of a new batch on arrival, lot verification testing may not be needed.” [15]. Low- and middle-income countries could significantly expedite access to safe and effective IVDs if they follow the recommended risk-based approach. As explained by WHO, the manufacturer has the primary responsibility for demonstrating that IVDs conform to the essential principles of safety and performance, quality requirements and all applicable national laws and regulations [2]. Under the manufacturer’s QMS, this includes any testing and documentation, all of which are subject to auditing and review by the NRA. All such testing forms the basis for the manufacturer’s declaration of conformity. Therefore, as part of their QMS, manufacturers have in place effective lot release procedures to ensure performance characteristics are maintained for each lot of products manufactured. Lot release testing should thus ensure that where appropriate, the sensitivity and specificity, or other critical performance characteristics remain unchanged for each lot by the testing of sufficient numbers of relevant samples. As a result, manufacturers not only produce the necessary data to secure market approval, but they are also bound by their QMS to ensure the accuracy of each lot produced. Many IVD products also have on board controls that offer additional evidence of lot performance at the user site or laboratory. Lastly, evidence of the QMS and adherence to it are inspected routinely (e.g., regulators, WHO, Medical Device Single Audit Program, or ISO 13485).
- **Labelling**. Country-specific labelling requirements add enormous complexity and cost to the manufacturer’s labelling process and can also act as a barrier to access. International standards exist for labelling and WHO recommends keeping country-specific labelling to the least burdensome [2].
- **Electronic submissions and electronic instructions for use**, including for consumer products, is the way of the future. As technology continues to develop, as well as smart processes like regulatory reliance [16], electronic submissions and harmonized dossiers will most certainly be key.
- **Overall development cost**. Development and commercialization of IVDs typically involve multiple phases: concept; feasibility/planning; design/development; validation; and approval/launch [17]. The process is lengthy, uncertain and costly, with one study estimating that the typical high-risk diagnostic tests (such as those for HIV) cost USD 94 million to advance from idea to market [18]. Market dynamics for diagnostics are distinct from those for medicines and vaccines. With profit margins for diagnostics tending to be lower, effective regulation of diagnostics, including those for HIV, requires a careful balancing as excessively onerous and duplicative requirements are a significant deterrent to marketing of diagnostic tools that have already been vetted and approved by “mature regulatory” agencies.

The overall lack of global or even regional convergence adds to regulatory complexity, increases cost, makes poor use of regulator resources, and acts as a barrier to access. There are National Medicines Regulatory Authorities or administrative entities in African countries, except for the Sahrawi Arab Democratic Republic, that carry out all or some aspects of medicine regulation [19], but their organizational set-up, regulation and functionality are highly variable and can differ from country to country. A three-country review in East Africa found considerable inconsistencies and ambiguities with respect to the regulation of medical devices, with some countries assigning responsibility to multiple agencies [8].

According to one interviewee, the situation is not unique to the African continent. In Southeast Asia, regulatory bodies responsible for medical device registration also differ in each country. The lack of a common regional framework and guidelines for Southeast Asia makes the process complicated for manufacturers who wish to register a product regionally as registration entails that a product approved in one Southeast Asia country undergoes mandatory clinical studies in another country.

The complexity and diversity in regulatory requirements and interactions add an enormous amount of complexity to the regulatory process for the same IVD product that is already approved in more mature markets (for example, the United States of America or the European Union). Due to the costs and effort required to meet country-specific registration requirements, companies in some cases may entirely forego seeking regulatory approval in smaller, low-income country markets for products with a low profit margin or in settings where there is low prevalence of the disease addressed by the new product. At times, companies are perceived externally as “neglecting” or “abandoning” those in need. Like other stakeholders, manufacturers operate within the limitations of finite resources.

In situations where demand exceeds capacity, which is often the case, companies, like national programmes or international donor agencies, must strategically allocate resources to maximize their positive impact. Low- and middle-income countries, through the implementation of regulations converging towards international best practices, adoption of global standards, and establishment of recognition or reliance mechanisms, could expedite access to advanced products used in clinical settings by high income countries, potentially within days or months. This would significantly improve access to diagnostics, expedite treatment, help improve health outcomes and contain increasing healthcare cost pressures.

#### The importance of reliance.

Reliance, including recognition, is a key regulatory tool recommended by WHO and adopted by both “mature” and developing regulators. Reliance is a strategy for efficiently utilizing resources, building regulatory expertise and capacity, and accelerating access to safe and effective, quality-assured medical products, no matter the countries’ regulatory “maturity”. Overall, reliance signifies an efficient, smart way to regulate in the modern environment and “does not represent a less stringent form of regulatory oversight or outsourcing of regulatory mandates, nor does it compromise independence. On the contrary, a decision to “regulate through reliance” is the hallmark of a modern and efficient regulatory authority.” Reliance is not a lesser form of regulatory oversight, but rather a strategy for making the best use of the available resources in any setting. Reliance allows the allocation of resources to other regulatory functions, such as vigilance and post-authorization activities, thereby increasing the effectiveness of local regulatory oversight [16].

Approval by a stringent regulator (for MD/IVDs, this is generally the original Global Harmonization Task Force, or GHTF, countries [20]) is widely regarded as the gold standard for regulatory oversight. However, approval by a stringent regulator authorizes a product to be marketed only in the agency’s jurisdiction. Thus, market authorization of a product by the U.S. Food and Drug Administration (FDA), for example, permits marketing in the United States only. Reliance on another jurisdiction, for example, the FDA, accelerates the regulatory approval process in another country. WHO recommends relying on regulators that have an equally stringent or more stringent regulatory framework. In some cases, such as mutual reliance or joint review, a confidentiality agreement may be needed.

That said, agreements are not required for all forms of reliance (for example, unilateral or reliance on Medical Device Single Audit Program, or MDSAP, inspections). This provides jurisdictions with the utmost flexibility to implement the reliance pathway that is best suited to their needs. Regulatory authorities, such as in Australia [21], Singapore [22] and Thailand [23], have had effective reliance pathways for some time and report improvement in regulatory review and access.

Expert opinions on the regulation of medicines, vaccines and diagnostics consistently emphasize the importance of reliance on, and recognition of, regulatory approvals by respected authorities [24–27]. This approach could help avoid potential delays associated with regulatory processes by enabling national authorities to accelerate approvals (including pre-market inspections) of diagnostic technologies that have already been rigorously reviewed. Willingness to adopt reliance is increasing, including in low- and middle-income countries, and regulators are collaborating more now than ever to learn how to operationalize reliance.

Informants also suggested that prioritization of a novel IVD by a major donor, such as the Global Fund for AIDS Tuberculosis and Malaria (GFATM) or the United States President’s Emergency Plan for AIDS Relief (PEPFAR), can also aid in expediting registration. One interesting example of a mechanism with potential to catalyse such access is the Expert Review Panel for Diagnostics system utilized by the GFATM and Unitaid and administered by the WHO. This initiative was specifically created to expedite access to critical Dx tools of public health significance, which are yet to obtain WHO prequalification (PQ) or Stringent Regulatory Authority (SRA) (GHTF-founding member) approval in a risk-mitigating and time-limited fashion. It must be noted, however, that this process has yet to meaningfully impact uptake of said priority diagnostic tools. Greater effort in communicating this programme and its benefits to national programs and grant recipients, particularly in the grant development phase but also throughout grant life-cycles presents a tremendous opportunity for improving access to new and critical tools. In cases where a product has been prequalified by WHO, countries can also benefit from WHO’s Collaborative Review procedure to achieve country approval within 90 days.

## WHO prequalification of HIV diagnostics

### WHO prequalification programme

While WHO is not a regulatory body, its programme for prequalifying medicines, vaccines and diagnostics plays a critical role in facilitating access to medical products in low- and middle-income countries. WHO PQ provides validation that diagnostics are appropriate for use in resource-limited settings. WHO prequalification is often used by UN agencies as a condition for the procurement of HIV IVDs and most major donors, although some donors allow for the purchase of products approved by a stringent regulator and/or subject to other approval processes, regardless of their WHO PQ status. With respect to diagnostics, most donors regard WHO PQ as equivalent to a stringent approval.

Since it began its prequalification programme for diagnostics in 2010, WHO has prequalified 68 HIV diagnostics (as of December 2024) [28]. These include rapid HIV diagnostic tests, nucleic acid tests for HIV diagnosis, HIV confirmatory tests, HIV self-tests and combination HIV/syphilis tests, as well as multiple platforms for viral load and CD4 monitoring. Most manufacturers of prequalified diagnostics are from high-income countries.

PQ involves a multi-step process, including a detailed product dossier and a product evaluation, according to the WHO’s Technical Standard and Protocol in a WHO-approved laboratory, to determine the products’ performance in resource-limited setting. WHO also conducts an inspection of the manufacturing site and a review of the labelling. Successful outcomes are required across all these steps before a product can be prequalified. More recently, the WHO has taken an executive decision to rely on Medical Devices Single Audit Program (MDSAP) inspections in lieu of a WHO specific inspection [29]. However, revised WHO guidance has not yet been published.

### Limitations of the WHO PQ programme

There is agreement that WHO PQ has improved access of underserved populations to quality-assured priority diagnostic tools [30]. An independent external evaluation found that WHO enabled access to medical products for 300-400 million people worldwide [31]. Yet, while WHO has become increasingly willing to improve the processes, much work remains to be done.

Manufacturers and other observers have highlighted various challenges associated with the PQ process. Although the average review time from submission to PQ is often cited as two to three years, several key informants indicated that the process can take significantly longer in practice. Given the WHO Collaborative Review Procedure’s dependency on PQ (see below), there is an opportunity to improve efficiency.

One suggested improvement is greater alignment with internationally recognized standards and a reduction in the use of WHO-specific standards and protocols. This could help streamline the process by minimizing the performance evaluation phase, which represents a substantial portion of the PQ process. If WHO were to align its standards and protocols with internationally accepted standards and protocols, as recommended in its Global Model Regulatory Framework (GMRF), it could more easily rely on approvals conducted by “mature regulators” – an approach also recommended in the GMRF.

Another factor influencing PQ timelines is the requirement for performance evaluations to take place in a WHO-accredited laboratory. While the purpose of PQ includes ensuring that products perform well in a lower-resource setting, five respondents (three from industry, one from WHO and one from low- and middle-income country NRA) reported that, in some cases, evaluation occurs in high-income countries like the United States or the United Kingdom. This has led to some concerns about alignment with WHO’s stated rationale for PQ.

WHO has indicated that it practices reliance on approvals from “mature regulators” through its abridged review pathway, as well as on MDSAP [32] inspections and ISO 13485 certifications. However, respondents reported that these reliance mechanisms have not significantly expedited the process. One industry interviewee noted that, despite leveraging the abridged review pathway, WHO was constrained by the specific requirements outlined in its respective standards and protocols. Another industry respondent shared that WHO required on-site inspections at two of its manufacturing sites even though the sites held MDSAP and ISO 13485 certifications.

One industry respondent suggested that reliance on “mature regulators” could, in certain cases, reduce the burden on PQ. For example, WHO recently announced PQ requirements for Tuberculosis products, despite these products already being available in the market for several years, including in low- and middle-income countries. Given WHO’s ongoing resource constraints—including limited funding and staffing— [33], its PQ programme faces challenges in keeping pace with an increasingly crowded product pipeline. Also, industry representatives have noted that PQ timelines are often less transparent compared to other regulatory processes. Previously, WHO published information tracking the progress of individual products through the PQ process, including dates indicating when specific milestones were achieved. This level of detailed reporting provided a useful way to evaluate PQ timelines and outcomes. Unfortunately, this information is no longer publicly available, which not only diminishes its potential to strengthen the important case for additional funding but also undermines transparency.

While PQ improvements are necessary, industry broadly acknowledges and appreciates WHO’s continued efforts to seek feedback and enhance its processes. For example, WHO’s PQ for IVDs has adopted a more integrated approach by conducting performance evaluations —often through outsourcing to expert laboratories— while the dossier is still being reviewed for completeness. Additionally, WHO has taken steps to implement reliance mechanisms via the abridged review and the collaborative procedure for accelerated registration programmes. Efforts have also been made to clarify dossier content requirements, enhance communication, and expand the list of approved laboratories for testing. One key informant noted that these changes have made PQ procedures clearer and more streamlined.

### Collaborative procedure for accelerated registration (CRP)

WHO has created procedures with the goal of expediting national registration of prequalified products [34], including the Collaborative Registration Procedure (CRP) for diagnostics, which was introduced in 2019. While the CRP depends on successful completion of PQ, it has the potential to accelerate access and reduce duplicative regulatory efforts.

To qualify for CRP, the product should remain fundamentally unchanged. This entails meeting WHO’s criteria, which include maintaining the same product name, regulatory version, product codes, manufacturing site and quality system. Additionally, the data on quality, safety and performance, must be consistent, as must the product design, components from the same suppliers, and the same information, labelling and packaging, and instructions for use and intended use. If these requirements are met and an agreement is in place between WHO, the relying country and the relevant regulatory authority, the CRP can yield country approval within 90 days.

However, while the CRP is designed to streamline regulatory approvals, its reliance and stringent eligibility criteria may present challenges for some manufacturers. Given the variation in regulatory requirements across countries—particularly for labelling and instructions for use—it may be difficult for some products to fully align with CRP criteria. In addition, as manufacturing continues to globalize and diversify, especially since COVID-19, a product may be produced under the same quality system but at a different manufacturing site. Under current CRP requirements, this would impact eligibility.

Despite these considerations, early implementation has shown promising results. A pilot study of the CRPs for diagnostics in six African countries found that two of the countries successfully registered the prequalified WHO product within the targeted 90 days. As of February 2023, 26 countries had signed up to participate in the CRP for IVDs, reflecting growing interest in leveraging this approach to facilitate regulatory approvals [35].

### Mechanisms to accelerate IVD regulatory approval

Several initiatives are currently underway to promote regulatory convergence and accelerate approval of IVDs. However, sustained attention is required to advance these efforts toward the end goal of maintaining and improving quality assurance while expanding access.

### Capacity building/regulator training

The Medical Device Regulatory Convergence (MDRC) project, a public-private partnership between the U.S. Agency for International Development (USAID), the American National Standards Institute (ANSI) and implementation partners, plays a key role in strengthening regulatory oversight capacity in low- and middle-income countries. Through numerous sessions and workshops, the project focuses on building effective and efficient regulatory frameworks, promoting the adoption of internationally recognized standards, advancing conformity assessment procedures and fostering private sector engagement [36]. These efforts are consistently supported by stringent regulators and the WHO, ensuring alignment with global best practices.

The Global Medical Technology Alliance (GMTA) recently published a white paper on global convergence and reliance [37]. The paper outlines core tenets for an effective, efficient and sustainable regulatory framework. The paper highlights the importance of “smart regulation”, which includes mechanisms such as regulatory reliance and recognition to streamline approval processes while maintaining high standards for safety and efficacy.

Also, the African Society for Laboratory Medicine (ASLM), founded in 2011, has rapidly become an important contributor to increased laboratory expertise in central, eastern, southern and western Africa. ASLM supported the creation of the Diagnostic Evidence Lab [38], which provides NRAs, national reference laboratory and other stakeholders with key data from published studies on the performance of IVDs for HIV. By making this data readily accessible, the initiative aims to reduce the time required for in-country validation.

### Harmonization & convergence

In place of broadly varying regulatory processes and approval criteria, regulatory convergence, reliance, and collaboration are the way of our regulatory future. For many years, the IMDRF has been a key driver of regulatory convergence. In 2024, IMDRF held two sessions specifically focused on regulatory reliance and committed to publishing a Reliance Playbook, which will serve as a key resource for jurisdictions looking to implement a reliance pathway. IMDRF has also supported global convergence for years and has published a range of guidelines to enable greater convergence [19]. Mandated by Resolution WHA 67.20, WHO plays a central role in strengthening regulatory systems. Through its Regulatory Systems Strengthening programme, WHO supports countries in developing more robust regulatory frameworks [39,40]. Furthermore, WHO’s Global Model Regulatory Framework references much of the IMDRF guidance, demonstrating a shared commitment to advancing global convergence.

In some regions, neighbouring countries have sometimes collaborated to harmonize regulatory approaches. For example, the European Union (EU) has long operated under a process of EU-wide regulation of IVDs, where products attaining a Conformité Européenne -IVD or CE-IVD mark are recognized across all member states. With the implementation of the *In Vitro* Diagnostic Regulation (IVDR) in 2022 [41], which introduced increased regulatory stringency, all CE-IVD marked products are now considered to have undergone stringent regulatory approval, regardless of their risk classification.

On the African continent, the Pan-African Harmonization Working Party, founded in 2012, has been instrumental in promoting convergence of regulatory approaches [42]. With support from WHO, regulators in Botswana, Namibia, Zambia and Zimbabwe have launched the ZaZiBona initiative to expedite the review and approval of medicines and vaccines [43]. Further advancing this effort, African countries joined together in 2021 to establish the African Medicines Agency (AMA) with the aim of reducing the time required for validated medicines to reach healthcare clients who need them. The African Union is currently operationalizing the AMA, with the goal of developing a continental regulatory reliance framework that will be piloted in eastern Africa [44]. In the Asia-Pacific region, the COVID-19 pandemic has reinforced the value of networking, collaboration, interdependence and acknowledgement among NRAs across the region [45].

## Conclusion and recommendations

While broad efforts are needed to strengthen laboratory and healthcare capacities, converging regulation based on reliance, recognition and adherence to internationally recognized standards offers a practical pathway to expediting access to safe and effective diagnostics, including HIV tests. Table 2 outlines recommendations tailored for key stakeholders in the field. The objective is to enhance regulatory capacity by embracing international best practices. Improving regulatory processes is a long-term and continuous initiative, demanding the sustained engagement and collaboration of all stakeholders. While trade agreements and international regulatory convergence can improve diagnostic availability and access across countries, strengthening domestic policies remains essential. Although the challenge is significant, the anticipated benefits extend beyond regulatory frameworks, promising significant advantages for global public health and, most importantly, end users and patients.

**Table 2:** Recommendation for key stakeholders involved in the regulation of HIV *in vitro* diagnostics.

Stakeholders	Recommendations
Manufacturers, regulators	Continue to build regulatory capacity based on international best practice (IMDRF, WHO, GMRF) and standards (for example, ISO).
Regulators	Implement smart regulation, including regulatory reliance or recognition through the total product lifecycle.
Regulators	Digitize regulator processes to allow electronic submissions, change requests and signatures.
Countries	Allow aid programmes to use funding to purchase either WHO prequalified products or products approved by a stringent regulator.
WHO	Implement recognition of stringent approvals, changes and inspections. Allow bridging studies or an alternative to supplement stringent approval when scientifically justifiable. Publish PQ reports outlining cycle times for major milestones to strengthen transparency. Align WHO Technical Standards and protocols with internationally accepted standards unless scientific justification is publicly provided. Where full PQ is necessary, allow the use of any accredited laboratory that meets the requirements of ISO/IEC 17025 General Requirements for the Competence of Testing and Calibration Laboratories. Implement a risk-based approach to review product changes. Only changes that significantly impact safety and effectiveness should require review (unless WHO can practice recognition on NRA approvals). Once these improvements have been made, assess the important case for additional funding of PQ.

## References

[1] Kelly-Cirino CD, Nkengasong J, Kettler H, Tongio I, Gay-Andrieu F, Escadafal C, et al. Importance of diagnostics in epidemic and pandemic preparedness. BMJ Glob Health. 2019;4(Suppl 2):e001179. doi: 10.1136/bmjgh-2018-001179 30815287 PMC6362765

[2] World Health Organization. WHO global model regulatory framework for medical devices including in vitro diagnostic medical devices 2017 [cited 2 August 2023]. Available from: https://apps.who.int/iris/handle/10665/255177

[3] Fleming KA, Horton S, Wilson ML, Atun R, DeStigter K, Flanigan J, et al. The Lancet Commission on diagnostics: transforming access to diagnostics. Lancet. 2021;398(10315):1997–2050. doi: 10.1016/S0140-6736(21)00673-5 34626542 PMC8494468

[4] Yadav H, Shah D, Sayed S, Horton S, Schroeder LF. Availability of essential diagnostics in ten low-income and middle-income countries: results from national health facility surveys. Lancet Glob Health. 2021;9(11):e1553–60. doi: 10.1016/S2214-109X(21)00442-3 34626546 PMC8526361

[5] McNerney R, Sollis K, Peeling RW. Improving access to new diagnostics through harmonised regulation: priorities for action. Afr J Lab Med. 2014;3(1):123. doi: 10.4102/ajlm.v3i1.123 29043177 PMC5637758

[6] World Health Organization. Strengthening diagnostics capacity. 76th World Health Assembly 2023 [cited 2 August 2023]. Available from: https://apps.who.int/gb/ebwha/pdf_files/WHA76/A76_R5-en.pdf

[7] Morin S, Bazarova N, Jacon P, Vella S. The manufacturers’ perspective on World Health Organization prequalification of in vitro diagnostics. Clinical Infectious Diseases. 2018;66(3):301–5. 29020182 10.1093/cid/cix719 PMC5848238

[8] Hubner S, Maloney C, Phillips SD, Doshi P, Mugaga J, Ssekitoleko RT, et al. The Evolving Landscape of Medical Device Regulation in East, Central, and Southern Africa. Glob Health Sci Pract. 2021;9(1):136–48. doi: 10.9745/GHSP-D-20-00578 33764886 PMC8087432

[9] De Maria C, Di Pietro L, Díaz Lantada A, Madete J, Makobore P, Mridha M. Safe innovation: On medical device legislation in Europe and Africa. Health Policy and Technology. 2018;7(1):156–65.

[10] Medical Device Coordination Group (MDCG). Guidance on Classification Rules for in vitro Diagnostic Medical Devices under Regulation (EU) 2017/746 2023 [cited Accessed 10 April 2023]. Available from: https://health.ec.europa.eu/document/download/12f9756a-1e0d-4aed-9783-d948553f1705_en

[11] World Health Organization. WHO Global Benchmarking Tools (GBT) for evaluation of national regulatory systems [cited 31 January 2025]. Available from https://www.who.int/tools/global-benchmarking-tools

[12] Marrakesh Agreement. World Trade Organisation. 1994 [cited Accessed 25 January 2024.]. Available from https://www.wto.org/english/docs_e/legal_e/17-tbt_e.htm

[13] In Vitro Diagnostic Device Regulatory Submission Table of Contents (IVD ToC). International Medical Device Regulators Forum 2023 [cited 25 January 2024]. Available from: https://www.imdrf.org/consultations/vitro-diagnostic-device-regulatory-submission-table-contents-ivd-toc

[14] Health Canada and US Federal Drug an Administration. eSTAR Pilot 2023 [cited 25 January 2024]. Available from: https://www.fda.gov/medical-devices/how-study-and-market-your-device/health-canada-and-fda-estar-pilot

[15] World Health Organization. WHO Global model regulatory framework for medical devices including in vitro diagnostic medical devices (GMRF). [cited 31 January 2025]. Available from: https://cdn.who.int/media/docs/default-source/biologicals/bs-2022.2425_global-model-regulatory-framework-for-medical-devices-including-ivds_11-july-2022-dl_14-july.pdf?sfvrsn=28d107c5_1

[16] World Health Organization. WHO Expert Committee on Specifications for Pharmaceutical Preparations: fifty-fifth report 2021 [cited 21 September 2023]. Available form: https://iris.who.int/handle/10665/340323

[17] Wang L, Xu W, Wang B, Si X, Li S. Methods and Advances in the Design, Testing and Development of In Vitro Diagnostic Instruments. Processes. 2023;11(2):403. doi: 10.3390/pr11020403

[18] Steinberg D, Horwitz G, Zohar D. Building a business model in digital medicine. Nat Biotechnol. 2015;33(9):910–20. doi: 10.1038/nbt.3339 26348957

[19] Ndomondo-Sigonda M, Miot J, Naidoo S, Dodoo A, Kaale E. Medicines Regulation in Africa: Current State and Opportunities. Pharmaceut Med. 2017;31(6):383–97. doi: 10.1007/s40290-017-0210-x 29200865 PMC5691122

[20] International Medical Device Regulators Forum [cited 25 January 2024]. Available from: https://www.imdrf.org/ghtf

[21] Therapeutic Goods Administration. Use of market authorisation evidence from comparable overseas regulators/ assessment bodies for medical devices (including IVDs) 2023 [cited 10 April 2024]. Available from: https://www.tga.gov.au/resources/resource/guidance/use-market-authorisation-evidence-comparable-overseas-regulators-and-assessment-bodies-medical-devices-including-ivds

[22] Health Sciences Authority. Register Class B medical device via abridged route 2023 [cited 9 February 2024]. Available from: https://www.hsa.gov.sg/medical-devices/registration/guides/class-b-abridged-registration

[23] Thai FDA. Medical Device Regulations in Thailand. Device Registration Pathways [cited 9 February 2024]. Available from: https://www.artixio.com/post/medical-device-regulations-in-thailand-thai-fda-device-registration-pathways

[24] Mugambi ML, Peter T, F Martins S, Giachetti C. How to implement new diagnostic products in low-resource settings: an end-to-end framework. BMJ Glob Health. 2018;3(6):e000914. doi: 10.1136/bmjgh-2018-000914 30498586 PMC6254739

[25] Doerr P, Valentin M, Nakashima N, Orphanos N, Santos G, Balkamos G, et al. Reliance: a smarter way of regulating medical products - The IPRP survey. Expert Rev Clin Pharmacol. 2021;14(2):173–7. doi: 10.1080/17512433.2021.1865798 33355025

[26] Xu M, Zhang L, Feng X, Zhang Z, Huang Y. Regulatory reliance for convergence and harmonisation in the medical device space in Asia-Pacific. BMJ Glob Health. 2022;7(8):e009798. doi: 10.1136/bmjgh-2022-009798 35985696 PMC9395591

[27] Saint-Raymond A, Valentin M, Nakashima N, Orphanos N, Santos G, Balkamos G, et al. Reliance is key to effective access and oversight of medical products in case of public health emergencies. Expert Rev Clin Pharmacol. 2022;15(7):805–10. doi: 10.1080/17512433.2022.2088503 35945703

[28] World Health Organization. WHO list of prequalified in vitro diagnostic products [cited 28 February 2025]. Available from: https://extranet.who.int/prequal/sites/default/files/document_files/list-of-prequalified-in-vitro-diagnostic-products_3.pdf

[29] World Health Organization. Enhanced reliance in WHO Prequalification of IVDs [cited 31 January 2025]. Available from: https://extranet.who.int/prequal/news/enhanced-reliance-who-prequalification-ivds

[30] ’t Hoen EFM, Hogerzeil HV, Quick JD, Sillo HB. A quiet revolution in global public health: The World Health Organization’s Prequalification of Medicines Programme. J Public Health Policy. 2014;35(2):137–61. doi: 10.1057/jphp.2013.53 24430804

[31] World Health Organization. Impact assessment of WHO Prequalification and systems supporting activities 2019 [cited 28 July 2023]. Available from https://www.who.int/publications/m/item/impact-assessment-of-who-prequalification-and-systems-supporting-activities

[32] US Federal Drug an Administration. Medical Device Single Audit Program (MDSAP) 2023 [cited 25 January 2024]. Available from: https://www.fda.gov/medical-devices/cdrh-international-affairs/medical-device-single-audit-program-mdsap

[33] Reuters. Under-funded WHO seeks ‘reinforced’ role in global health at key meeting [cited 1 August 2023]. Available from: https://www.reuters.com/business/healthcare-pharmaceuticals/under-funded-who-seeks-reinforced-role-global-health-key-meeting-2023-01-30

[34] World Health Organization. Collaborative procedure between the WHO and NRA’s in the assessment and accelerated national registration of WHO-prequalified IVD’s - Annex 4 2021 [cited 3 November 2023]. Available from: doi: https://www.who.int/publications/m/item/collaborative-procedure-between-the-who-and-nra-s-in-the-assessment-and-accelerated-national-registration-of-who-prequalified-ivd-s-annex4

[35] International Medical Device Regulators Forum. WHO Update by Irena Prat, Team Lead - IVDs assessment Reulation and Prequalification Department, World Health Organization [cited 2025 March 19]. Available from: doi: https://www.imdrf.org/sites/default/files/docs/imdrf/final/meetings/imdrf-meet-200921-singapore-webconference-20.pdf?utm_source=chatgpt.com

[36] Medical Device Regulatory Convergence Project and Standards Alliance. Standards Alliance Phase 2 COVID-19 Medical Device Regulatory Convergence Project (MDRC) [cited 25 January 2024]. Available from: https://www.standardsalliance-mdrc.org/mdrc-project-and-standards-alliance

[37] Global Medical Technology Alliance. The Need to Advance Global Convergence and Regulatory Reliance to Accelerate Access to Medical Technology [cited 29 January 2024]. Available from: https://www.globalmedicaltechnologyalliance.org/papers/GMTA%20Global%20Harmonization%20of%20Medical%20Technology%20Regulations.pdf

[38] African Society for Laboratory Medicine. Diagnostic Evidence Hub [cited 2 August 2023]. Available from: https://aslm.org/diagnostic-hub

[39] World Health Assembly. Regulatory system strengthening for medical products - WHA67.20. 2014 [cited 12 March 2024]. Available from: https://www.who.int/publications-detail-redirect/A67_R20

[40] World Health Organization. Regulatory systems strengthening [cited 12 March 2024]. Available from: https://www.who.int/teams/regulation-prequalification/regulation-and-safety/rss

[41] European Parliament, Council of the European Union. Regulation (EU) 2022/112 of the European parliament and of the council of 25 January 2022 [cited 29 January 2024]. Available from https://eur-ex.europa.eu/eli/reg/2022/112/oj

[42] McNerney R, Peeling R. Regulatory in vitro diagnostics landscape in Africa: Update on regional activities. Clinical Infectious Diseases. 2015;61(Supplement 1):S135–40. 10.1093/cid/civ553 26409274

[43] Sithole T, Mahlangu G, Walker S, Salek S. Regulatory Authority Evaluation of the Effectiveness and Efficiency of the ZaZiBoNa Collaborative Medicines Registration Initiative: The Way Forward. Front Med (Lausanne). 2022;9:898743. doi: 10.3389/fmed.2022.898743 35547217 PMC9082034

[44] African Union Development Agency. AMRH Updates Sept-Oct 2023 [cited 25 January 2024]. Available from: https://www.nepad.org/publication/amrh-updates-sept-oct-2023

[45] Lacey S, Mitchell A. Regulatory cooperation for vaccines: The Asia-Pacific and beyond. Asian Int Studies Rev. 2023;24(1):74–102.

